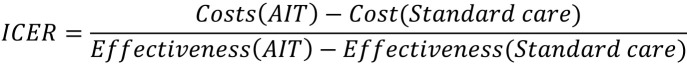# Clinical endpoints in allergen immunotherapy: State of the art 2022 

**DOI:** 10.5414/ALX02334E

**Published:** 2023-03-01

**Authors:** Roy Gerth van Wijk, Ludger Klimek, Oliver Pfaar

**Affiliations:** 1 Section of Allergology and Clinical Immunology, Department of Internal Medicine, Erasmus MC, Rotterdam, the Netherlands,; 2 Center for Rhinology and Allergology, Wiesbaden, and; 3 Department of Otorhinolaryngology, Head and Neck Surgery, Section of Rhinology and Allergy, University Hospital Marburg, Philipps-Universität Marburg, Marburg, Germany

**Keywords:** allergen immunotherapy, endpoints, rhinitis, asthma, real-life studies, cost-effectiveness

## Abstract

110 years after the classical study by Noon, numerous studies have confirmed the efficacy of allergen immunotherapy. A variety of clinical endpoints have been used in these trials. This review gives an overview of clinical endpoints for randomized clinical trials on allergen immunotherapy (AIT) in rhinitis and asthma. In addition, real-life studies have been carried out with the same kind of endpoints. In general, AIT studies are characterized by a lack of standardized and validated outcome measures. For allergic rhinoconjunctivitis, digital tools have been developed to monitor patients. Such tools are particularly useful to obtain real-world evidence for AIT. Finally, well-accepted outcome measures are available for cost-effectiveness studies.

## Introduction 

In 1911, Noon [1] was the first to describe the effectiveness of grass pollen immunotherapy in a hay fever patient. To assess the effect of immunotherapy, he studied the sensitivity of the conjunctivae to allergen extract during treatment. After this study, practical experience, case reports, and case series supported the effectiveness of allergen immunotherapy (AIT) treatment. Frankland and Augustin published the first placebo-controlled study in 1954 [2]. In this study, the effect of AIT was measured by a global patient assessment. Patients reported the effects of treatment as excellent, good, moderate, or poor. Frankland and Augustin used this outcome measure for both seasonal hay fever and pollen asthma. Since then, numerous randomized controlled trials (RCTs) have been published. 

However, the absence of generally accepted outcome measures led to a substantial heterogeneity in primary and secondary endpoints for AIT trials. To overcome the lack of standardization, a comprehensive EAACI (European Academy of Allergy & Clinical Immunology) position paper on clinical outcomes in AIT trials for allergic rhinitis was published in 2014 [3]. Fewer studies have been published on the effects of AIT on asthma. Also, asthma studies have been characterized by a wide variation of outcome measures. 

In recent years, there has been an increasing awareness that the acceptance of AIT in daily practice also depends on its effectiveness in daily practice. From that perspective, there is an increasing focus on “real-life” studies with their own methodology. Information and technology (ICT) tools have been developed that might be helpful in assessing effectiveness in real-life studies. Finally, the effects of treatment must outweigh the costs. Cost-effectiveness studies are required by regulatory agencies to assess the economically added value compared to standard care. Specific endpoints have been developed to evaluate the economic benefit of new treatments. 

There is a need for well-accepted and standardized tools to measure clinical efficacy of AIT. This review aims to present an overview of current clinical endpoints for AIT studies in rhinitis and asthma. 

## Clinical endpoints in rhinoconjunctivitis studies 

Validated clinical endpoints such as symptom/medication scores are needed to assess the efficacy of any type of allergic rhinitis treatment [3, 4, 5, 6]. 

AIT is just one example of many in this regard. However, because AIT studies are very complex due to the usually longer study duration, dependence on allergen exposure, and use of rescue medication as adjunctive therapy, an EAACI task force has collected all available clinical endpoints and made recommendations for their use in AIT studies [3]. The combined symptom/medication score (CSMS), recommended here as the primary outcome parameter, has been used in numerous AIT trials [7, 8, 9, 10, 11]. This CSMS of the EAACI can better discriminate between active and placebo treatment than the separate evaluations of symptom and medication scores [12]. However, the CSMS has not yet been prospectively validated [6]. Mobile health-based approaches are currently helping to address this issue, for example in the MASK-air project (Mobile Airways Sentinel networK), which uses an app with a questionnaire in which allergic symptoms and medication use are assessed daily by the patient [13, 14, 15]. In a recent MASK-air study, a modified CSMS was analyzed [16]. Here, in the absence of a true gold standard, validation of this new CSMS was performed by comparison with endpoints such as work productivity and quality of life [17, 18]. The same approach has been followed in another recent trial of the group of MASK-air [19]. In this, more than 317,000 reported days from 17,780 users in 25 countries have been investigated regarding the correlation of the modified CSMS with the three endpoint measures work-productivity (WPAI-AS), quality-of-life (EQ-5D), and control of allergic diseases (CARAT). The analysis revealed a medium-high validity, reliability, and accuracy of the modified CSMS recommending this endpoint (CMMS) for primary endpoint analyses of future rhinitis trials. 

Secondary endpoints in AIT studies include subjective parameters such as visual analog scales (VAS) for a wide variety of parameters, health-related quality of life (HRQL), well and severe days, global assessment of patient satisfaction, and rhinitis control measures [3, 8, 9, 20]. VAS are well validated and widely used today [20, 21]. 

In particular, in the current COVID-19 pandemic, they have proven to be meaningful parameters that are easy to collect and valid, especially in telemedicine applications [22, 23, 24, 25, 26]. Objective secondary endpoints also include assessment by nasal or conjunctival provocation testing or by exposure in allergen exposure chambers [27, 28, 29]. However, further technical and clinical validation is required to extrapolate the clinical effects of provocation tests to the situation under natural allergen exposure before they are accepted as relevant for approval [29, 30, 31]. 

In terms of laboratory biomarkers, the measurement of antigen-specific IgG4-type antibodies in AIT studies is considered a classical parameter and other laboratory biomarkers are currently under discussion [32]. The clinical symptom improvements with AIT may also be accompanied by a reduction in the number of immunological cells such as mast cells, basophils, eosinophils, and type 2 lymphocytes in nasal and bronchial mucosa [32]. Thus, production of blocking IgG/IgG4 antibodies can inhibit IgE-dependent activation, which may be triggered by high-affinity IgE receptors (FcεRI) on mast cells and basophils, as well as via low-affinity IgE receptors (FcεRII) on B cells [32]. Deletion or anergy of antigen-specific T cells, induction of antigen-specific regulatory T cells, or induction of TH1 cells occurs concomitantly [32]. 

## Clinical endpoints in asthma studies 

Whereas recommendations for clinical endpoints in AIT are available for allergic rhinoconjunctivitis [3], such recommendations for asthma research have not been established. However, systematic reviews and meta-analyses give a good overview of available outcome measures [33, 34, 35]. 

Symptom scores have been most often used, followed by medication scores and, to a lesser extent, combined symptom/medication scores [34]. In this EAACI systematic review [34], these outcomes were used as primary endpoints. For a meta-analysis on the efficacy of sublingual immunotherapy, Fontescue et al. [35] considered the following primary outcomes: 1) exacerbation requiring emergency department (ED) visit or hospitalization (participants with at least one), 2) quality of life as measured with a validated scale, e.g., Asthma Quality of Life Questionnaire, and 3) serious adverse events. Secondary outcomes comprised 1) asthma symptom scores (measured on a validated scale, e.g., Asthma Control Questionnaire), 2) exacerbations requiring systemic corticosteroids, 3) response to provocation tests, 4) required dose of inhaled corticosteroids. The authors selected these outcomes as the most endpoints important for people with asthma. 

Although the choice for primary outcomes may differ in meta-analyses compared to clinical trials, the difference in approach between the latter two systematic reviews clearly reflects the lack of a well-accepted method in asthma AIT trials. 

A meta-analysis only focusing on subcutaneous immunotherapy comprised symptom scores, medication scores, lung function (including peak expiratory flow, forced expiratory volume in one second, and thoracic gas volume), nonspecific and allergen specific bronchial hyperreactivity [33]. 

Table 1 summarizes the different endpoints in AIT trials and their use in meta-analyses. Whereas symptom scores and lung function measures have been the endpoints of choice in the past [33], nowadays emphasis is being placed on exacerbations and validated scores such as the asthma control and quality of life scores [35]. Heterogeneity and lack of validation of symptom and medication scores are important disadvantages [35]. Despite these disadvantages, pooled estimates of symptom and medication scores successfully confirmed efficacy of AIT in asthma [33, 34]. The use of exacerbations, asthma control and asthma quality of life scores, and steroid reduction as endpoints are in line with the trial designs of studies with asthma biologics [36]. However, monitoring of ED visits and hospital admission for exacerbations is useful for assessing patients with severe or difficult to treat asthma, conditions that are eligible for treatment with biologics but not with AIT. The approach to establish the risk of an asthma exacerbation after tapering off corticosteroids may be more appropriate [37, 38]. However, it is still a matter of debate which endpoints are the most optimal primary outcomes in AIT asthma trials. 

## Endpoints in real-life studies 

The efficacy of AIT has been confirmed by numerous RCTs. RCTs have a robust design with strict inclusion and exclusion criteria for participants, uniform procedures for using concomitant medications, and regular monitoring of patients. These conditions are ideal for obtaining registration and market authorization of new AIT products. However, the study populations in RCTs may not resemble the average patient population in daily practice. The latter group may be more variable in demographic and clinical characteristics. Moreover, the expensive RCTs are less appropriate to assess long-term safety, efficacy, and patient compliance in daily life. 

In recent years, real-life studies have been carried out to overcome the drawbacks of RCTs. A recent systematic overview of 18 real-life studies [39] shows that the design of the AIT real-life studies may substantially vary in terms of duration, allergens, control population (if any) and outcome measures. Eight reported studies were prospective, the rest was retrospective (usually registry-based). Endpoints varied according to the aim of the studies and could be broadly divided into outcomes evaluating side effects, outcomes measuring reduction of medication and symptoms, and endpoints looking at patient adherence. It appears however, that standardization in endpoints is lacking. For instance, to assess clinical efficacy combined asthma/rhinitis medication scores and scores, symptom and medication scores, reduction of medication prescriptions, the risk of incident asthma, and percentage of medication-free subjects have been used. 

A systematic review of 14 observational comparative effectiveness studies in AIT reported a consistent positive effect of AIT over pharmacotherapy, but the magnitude of this benefit was difficult to estimate, owing to different scales and scoring systems in the studies [40]. This review identified flaws in the design and methodology and confounders in the majority of studies. 

In spite of these drawbacks encountered in real-life studies, the importance of real-world data and real-world evidence to complement the results of RCTs has been underlined by the Food and Drug Agency (FDA) [41]. The FDA states that real-world data can be generated from different study designs and analyses such as RCTs, large simple trials, pragmatic trials, and observational studies. 

Among other sources, mobile phones to monitor the patient’s symptoms may generate real-world evidence and overcomes the aforementioned lack of standardized clinical outcomes. In a proof-of-concept study using the MASK-air [16], global symptoms median VAS and median work VAS appeared to be lower in subjects treated with AIT compared to patients without AIT [42]. 

## Endpoints in cost-effectiveness analyses 

While AIT studies mainly try to determine the effectiveness of a treatment, cost-effectiveness analysis (CEA) aims to estimate the costs of a potential gain of treatment. Results from CEAs are usually reported as a single measure of economic benefit, the incremental cost-effectiveness ratio (ICER) [43]. The ICER for AIT can be expressed as[Fig Equation1]

CEA may be based on RCTs, but data and assumptions from meta-analyses, patient reports, questionnaires, available literature, and expert opinions can also be used [43, 44]. A variety of effectiveness outcomes have been reported in CEA studies to AIT: cost per number of patients approved and number of asthma cases avoided, cost per well day and symptom-free day, number of additional patients free from asthma symptoms, and costs per asthma case avoided [43]. It is, however, difficult to compare studies with this variation in measures. A standard effectiveness and most commonly used outcome is the quality-adjusted life year (QALY) with a weight of 1 representing optimal health. With this effectiveness outcome, policy makers can compare the cost-effectiveness of treatments across different diseases. The focus of a recent systematic review was to present results on QALYs [44]. Most QALY values were derived from the EuroQol five dimension questionnaire (EQ-5D), but a series of studies mapped other outcome measures such as symptom scores or the rhinitis quality of life questionnaire to QALYs. However, such conversions are not validated. This systematic overview from EAACI suggested that AIT for rhinitis and asthma is cost-effective, although the quality of included studies was low [44]. 

## Conclusion 

In the past decades, the efficacy of AIT has been further confirmed for both allergic rhinoconjunctivitis and asthma. However, study results are based on a variety of outcomes. For rhinitis studies, the CSMS is still the recommended primary outcome. Whereas in the past asthma trials were mainly assessed by symptom scores and lung function, nowadays other outcomes such as asthma control questionnaires, asthma exacerbations (especially after tapering off corticosteroids), or steroid reduction are more often used. However, it is still a matter of debate which endpoints are the most optimal outcome measures. 

Also research on real-life evidence points at a consistent beneficial effect of AIT. Real-life studies may offer valuable information not easily captured by classical placebo-controlled studies. Using MASK-air – a newly developed digital app to obtain real-life data – CSMS have been validated and put forward as a possible primary outcome. 

To analyze cost-effectiveness of AIT, well-accepted outcomes are available. Calculation of the QALY is the classical approach accepted by European authorities. Although less robust, studies point at cost-effectiveness for AIT in rhinitis and asthma. 

## Funding 

None. 

## Conflict of interest 

RGvW declares no conflict of interest.

LK has received research grants from Allergy Therapeutics/Bencard, Great Britain/Germany; ALK-Abelló, Denmark; Allergopharma, Germany; ASIT Biotech, Belgium; AstraZeneca, Sweden, Bionorica, Germany; Biomay, Austria, Boehringer Ingelheim, Germany, Circassia, USA; Stallergenes, France; Cytos, Switzerland; Curalogic, Denmark; HAL, Netherlands; Hartington, Spain; Lofarma, Italy; Viatris/Mylan, USA; Novartis, Switzerland, Leti, Spain; ROXALL, Germany; GlaxoSmithKline (GSK), Great Britain; Sanofi, France and/or has served on the speaker’s bureau or was consulting for the above mentioned pharmaceutical companies. LK is the current President of German Society of Allergology AeDA, Vice-President of the European Academy for Allergy and Clinical Immunology (EAACI), Vice-President of German Academy of Allergology and Environmental Medicine and Editor-in-Chief of AllergoJournal and AllergoJournal International. 

OP reports grants and/or personal fees from ALK-Abelló, Allergopharma, Stallergenes Greer, HAL Allergy Holding B.V./HAL Allergie GmbH, Bencard Allergie GmbH/Allergy Therapeutics, Lofarma, ASIT Biotech Tools S.A., Laboratorios LETI/LETI Pharma, GlaxoSmithKline, ROXALL Medizin, Novartis, Sanofi-Aventis and Sanofi-Genzyme, Med Update Europe GmbH, streamedup! GmbH, Pohl-Boskamp, Inmunotek S.L., John Wiley and Sons, AS, Paul-Martini-Stiftung (PMS), Regeneron Pharmaceuticals Inc., RG Aerztefortbildung, Institut für Disease Management, Springer GmbH, AstraZeneca, IQVIA Commercial, Ingress Health, Wort&Bild Verlag, Verlag ME, Procter&Gamble, ALTAMIRA, Meinhardt Congress GmbH, Deutsche Forschungsgemeinschaft, Thieme, Deutsche AllergieLiga e.V., AeDA, Alfried-Krupp Krankenhaus, Red Maple Trials Inc., all outside the submitted work. 

**Table 1.** Clinical endpoints in three meta-analyses of asthma trials (Cochrane SCIT analysis [33], EAACI systematic review [34], Cochrane SLIT analysis [35]).

**Clinical endpoints**	**Effect of AIT over placebo in asthma trials**	**Remarks**
Symptom scores	Large effect of AIT [34]; no meta-analysis possible [35]; significant effect in SCIT [33]	Heterogeneity; no validated scores
Medication scores	Large effect of AIT [34]; no meta-analysis possible [35]; significant effect in SCIT [33]	Heterogeneity; no validated scores
Symptom/medication scores	Small effect of AIT [34];	Small number of trials
Asthma control scores	No conclusion [34]	The study with the lowest ROB [34] did not show an effect [38]
Asthma quality of life scores	Large effect of SCIT; no conclusion for SLIT [34], no consistent pattern for SLIT [35]	The study with the lowest ROB [34] did not show an effect [38]
Exacerbations requiring emergency department visit or hospital admission	No conclusion because of a variety of methods [34]; 2 positive SLIT studies, very low evidence [35]	
Exacerbations requiring corticosteroids	No conclusion because of a variety of methods [34]. SLIT reduced the odds of experiencing an exacerbation with low certainty evidence [35]	The SCIT study with the lowest ROB [34] did not show an effect [45]. Two SLIT studies reported a positive effect of AIT on asthma exacerbations [34], one in the context of reducing the dose of ICS [38]. In a recent pediatric study – not included in these meta-analyses - SLIT HDM tablets were not able to reduce the risk of exacerbations [37].
Required dose of ICS	Effect of SLIT with low certainty evidence [35]	
FEV_1_, PEF	Effect of AIT [34]; no significant effect in SCIT [33]	
Non specific bronchial hyperreactivity	No clear effect of AIT [34]; effect of SLIT [35]; modest reduction in SCIT [33]	
Allergen specific bronchial hyperreactivity	Effect of SCIT [34]; significant reduction in SCIT [33];	Not practical, risk of late asthmatic reactions

SCIT = subcutaneous immunotherapy; EAACI = European Academy of Allergy & Clinical Immunology; SLIT = sublingual immunotherapy; AIT = allergen immunotherapy; ROB = risk of bias; ICS = inhaled corticosteroids; HDM = house dust mites; FEV_1_ = forced expiratory volume in one second; PEF = peak expiratory flow.

**Equation 1. Equation1:**